# Parental consent: A potential barrier for underage teens' participation in an mHealth mental health intervention

**DOI:** 10.1016/j.invent.2020.100328

**Published:** 2020-05-20

**Authors:** Patricia Cavazos-Rehg, Caroline Min, Ellen E. Fitzsimmons-Craft, Bria Savoy, Nina Kaiser, Raven Riordan, Melissa Krauss, Shaina Costello, Denise Wilfley

**Affiliations:** Department of Psychiatry, Washington University School of Medicine, St. Louis, MO, USA

## Abstract

**Purpose:**

We sought to examine whether underage adolescents displaying symptoms for a mental illness (i.e., an eating disorder) would be willing to obtain parental consent to participate in a study to test the efficacy of an evidence-based mobile mental health intervention targeting teens with eating disorders.

**Methods:**

The participants (*n* = 366) were 15 to 17 year-old English-speakers who post or follow social media accounts on Instagram that emphasize being thin as important or attractive. The participants were administered a survey through Qualtrics to assess eating disorder pathology, interest in trying an evidence-based mobile mental-health intervention, and comfort level with obtaining parental consent to partake in a research study about such an intervention.

**Results:**

About 85% of participants met clinical or subclinical criteria for an eating disorder; however, only 12% had received a treatment within the past six months. While 83% of participants were interested in trying a mobile health interventions app, only 35% indicated willingness to obtain parental consent to participate in a research study. The primary reasons presented for unwillingness to obtain consent included importance of retaining privacy and feeling that parents lack awareness or understanding about mental health issues.

**Conclusions:**

While barriers exist to obtaining treatment for eating disorders, a mobile intervention app may close some of these gaps. Many underage participants indicated interest in obtaining such treatment, yet only a third were willing to obtain parental consent. Future studies should investigate how to reduce these barriers to obtaining parental consent to facilitate teen access to research and mobile mental health treatment.

## Highlights

• Surveyed 15–17 year-olds posting or following Instagram content emphasizing being thin.
• 83% of participants were interested in trying a mobile health interventions app.
• Only 35% indicated willingness to obtain parental consent to participate in research.
• Reasons included privacy and perceived parental lack of mental health understanding.

## 1 Introduction

Digital interventions that are designed to achieve health objectives (i.e., mHealth) have been found to successfully involve adolescents in their own health care and expand health education in ways that advance health knowledge, behavior change, and improve medication adherence and disease management (Malbon and Romo, 2013; Militello et al., 2012; Seko et al., 2014). Moreover, the prominent use of smartphones among adolescents has led to an increase of health-related apps, games, and wearable devices targeted to this population, and about a quarter of adolescents report using these types of digital health tools (Wartella et al., 2016). While being cost-effective and efficient, digital interventions may also enable researchers to reach a more demographically inclusive sample including underage adolescents that are at-risk (i.e., exhibiting behavior that puts them at risk for imminent negative consequences) (Fenner et al., 2012). Moreover, emerging research supports the use of digital interventions to address serious mental illnesses, including depression and anxiety, among both adults and adolescents (Ebert et al., 2015; Hollis et al., 2017; Kazdin, 2019; Rooksby et al., 2015).

Similar to depression and anxiety, eating disorders (EDs) are serious mental illnesses associated with high comorbidity and mortality, and poor quality of life (Klump et al., 2009). EDs have the potential to harm every organ system and their health consequences are often more severe and/or manifest themselves to a greater degree among adolescents (Modan-Moses et al., 2003; Olmos et al., 2010). Digital interventions have demonstrated initial efficacy for helping adults with EDs (Fitzsimmons-Craft et al., 2019; Saekow et al., 2015) and Internet-delivered cognitive behavior therapy (ICBT) has shown promise in increasing the availability of effective psychological treatments for adolescents with psychiatric and somatic conditions, including those experiencing overeating and ED symptoms (Vigerland et al., 2016). However, there is a need for further exploration of the value of these tools among underage adolescents with EDs, which are highly prevalent, with roughly 13% of adolescents experiencing clinical and subclinical EDs (Stice et al., 2013; Swanson et al., 2011) and a peak age of onset during the teenage years (Volpe et al., 2016).

Digital interventions may be a viable method to reach adolescents with EDs especially given that these technologies are already well integrated into adolescent culture and can offer both privacy and accessibility (Berry and Lai, 2014; Murray et al., 2016). Although evidence from digital platforms supports Internet-based prevention and treatment programs for anxiety and depression, more extensive and rigorous research is warranted for additional mental health conditions, including EDs (Das et al., 2016). Research supporting the use of digital interventions designed specifically for adolescents with EDs is limited due in part to a mandate that requires minors under the age of 18 to receive parental permission in order to participate in mental health research (Liu et al., 2017). Underage adolescents with mental illnesses, including EDs, often feel stigmatized, lack awareness about their disorder, and/or seek privacy and independence from their parents (Balen et al., 2006); these are situations that can reduce adolescents' willingness to facilitate and/or allow for parental involvement if and when participation in a digital intervention has this requirement. Understanding if and how parental consent may act as a barrier to underage teens' participation in research of novel digital therapeutic tools for minors struggling with mental illness is critical to inform future directions for study and care. In identifying and taking steps to overcome this potential barrier, future studies may increase facilitation of teen and adolescent participation in accessible digital interventions and related research studies and enable the refinement of these interventions based on feedback directly from the population they aim to serve.

In the U.S., <20% of individuals with EDs, including adolescents, receive treatment (Forrest et al., 2017; Kazdin et al., 2017) and there is potential for digital intervention to address the remarkable treatment gap for adolescents with EDs (Kazdin et al., 2017). Previous studies with adolescents have been granted a parental waiver of consent due to the vulnerable nature of their adolescent research population (e.g., sexual minorities, substance use, eating disorders) (D'Souza et al., 2005; Reyes-Rodríguez et al., 2010; Rojas et al., 2008), including several studies utilizing text-based interventions and digital therapeutics (Schnall et al., 2015; Ybarra et al., 2016). Using a parental consent waiver has also been found to potentially reduce research costs, increase participant response rate, and expand data available to contribute to crucial adolescent health issues (Crane and Broome, 2017). Therefore, we wanted to determine adolescent perceptions of ED interventions and if rates of engagement with these potential treatments may possibly be reduced due to parental consent requirements. We aim to identify potential barriers such as parental consent in this study so that interventions (especially novel ones like a mHealth ED intervention) can be developed with feedback and input from teens from the start. Through an online survey of adolescents aged 15–17 years, this study seeks to understand the requirement for parental consent as a possible barrier to adolescents participating in research involving a mobile mental health app. Additionally, a qualitative analysis of open-ended responses further elucidates the concerns underlying their uncertainty about or unwillingness to allow researchers to contact their parents to obtain their parental consent for research purposes.

## 2 Methods

### 2.1 Participants and recruitment

Participants were recruited between February 13 and March 5, 2018 to participate in an online survey. English-speaking, 15–17 year-old persons living in the U.S. were recruited through ads on Instagram using keywords, such as “body image”, “body shape”, and “National Eating Disorders Association”. The goal of this method was to target individuals who displayed an interest in and/or followed Instagram accounts about EDs, body image, or ED-related topics. After clicking on the ads that described our recruitment into a study to learn about how social media impacts body image, individuals were directed to our study website, which contained a link to take an online eligibility survey. Eligibility requirements for participants included being aged 15–17 years, residing in the U.S., and *“either posting on social media or following social media accounts about eating, weight, and/or body image that emphasize being thin as important or attractive”.* Next, the eligible participants were presented the risks and benefits of the study (i.e., minimal risk of breach of confidentiality and no personal benefits beyond helping others benefit from what is learned), consented to participate, and were automatically forwarded to complete the online survey. The survey was created and administered through Qualtrics survey software (Qualtrics, Provo, UT) and was accessible by a computer or mobile device. These methods were approved by the Washington University institutional review board (IRB # 201611145) and a waiver of parental consent was granted given the minimal risks of the study and because the potential for our research to facilitate mental health treatment engagement for at-risk individuals outweighed the risks involved of not obtaining parental consent. Prior to entering the survey, participants were informed that this study consisted of a survey only and that no treatment would be directly provided. Participants were compensated with a $10 Amazon.com gift card for completing the survey.

The eligibility survey was accessed by 1535 individuals. Of the 606 eligible individuals, 600 consented to participate. Captcha was used to prevent machine responses and a Qualtrics feature helped prevent duplicate responses. In addition, the data were cleaned to remove low-quality survey responses (Bauermeister et al., 2012; Leiner, 2013) including 158 participants who did not progress through at least 40% of the survey, 36 who completed the survey in <8 min (lowest 10th percentile of survey completion time), 6 who were <15 years old, 3 participants with duplicate usernames, 30 who did not post or follow the content of interest, and 1 with contradictory responses. The final number of participants for analysis was 366.

### 2.2 Survey measures

Participants provided their demographic characteristics. Also, to evaluate the risk of having an ED based on DSM-5 categories (i.e., not at risk, at risk for an ED, possible anorexia nervosa (AN), bulimia nervosa (BN), binge eating disorder (BED), subclinical BN, subclinical BED, purging disorder, unspecified feeding or eating disorder (UFED)), the Stanford-Washington ED Screen (SWED) was used. The SWED is an online self-report tool that assesses ED pathology and risk with sensitivity and specificity for most diagnoses (Graham et al., 2018). To assess participants' quality of life, the Eating Disorders Quality of Life questionnaire (EDQOL), a validated 25-item scale to assess health-related quality of life that is associated with disordered eating (Engel et al., 2006), was used. Participants were asked if they had received treatment for any eating related problems in the past six months in order to determine their utilization of health care services. The presence of psychological comorbidities was evaluated using the Patient Health Questionnaire (PHQ-9), a nine-item instrument used to screen for depression severity based on DSM-IV diagnostic criteria (Kroenke et al., 2001), and the Generalized Anxiety Disorder 7-item scale (GAD-7), which was used to screen for anxiety severity (Spitzer et al., 2006). To determine ‘major depression’ and ‘GAD’, threshold scores of ≥10 on the PHQ-9 (sensitivity and specificity both 88%) (Kroenke et al., 2001) and ≥10 on the GAD-7 (sensitivity 89%, specificity 88%) (Kroenke et al., 2007) were used.

Respondents were asked about their interest in trying an evidence-based mobile mental health application (“app”) for EDs that included e-coaching. The app was described to respondents as a free, easy to use, highly interactive, and engaging mobile mental health resource and screenshots were provided of a sample app. Additionally, for respondents who expressed interest in this app, their willingness to allow researchers to contact their parents to obtain their consent for use of this intervention in a research study was subsequently assessed (i.e., “If we were going to test an app like this in a teen population, would you be willing to allow researchers to get your parent's permission for you to use the app (by contacting your parents through email and/or phone)?”). Answer choices to the latter question included ‘yes’, ‘no’, and ‘I'm not sure’. If respondents answered ‘yes’, they were directed to indicate the method through which they would feel comfortable with researchers contacting their parents; response choices were ‘email only’, ‘phone only’, or ‘either phone or email’. If respondents answered ‘no’ or ‘I'm not sure’, they were directed to open-ended questions to provide further explanation about their concerns about researchers contacting their parents for consent.

### 2.3 Analysis

SAS version 9.4 (SAS Institute, Cary, NC) was utilized for all statistical analyses. Descriptive statistics (percent, median, inter-quartile range) were used to explain the demographic and clinical characteristics of our sample. Among individuals who were interested in the mobile mental health app, respondents willing to allow researchers to contact parents to obtain parental consent versus those who would not or were unsure about obtaining parental consent were compared. Pearson Chi-square tests were used to compare categorical and Mann Whitney *U* tests were used to compare continuous variables. *P* < 0.05 was considered statistically significant.

For the analysis of qualitative responses to open-ended items querying reasons for an uncertainty about or unwillingness to allow researchers to contact parents to obtain parental consent, a combination of a deductive and inductive approach was used (Braun and Clarke, 2006). Through a deductive approach, this study's initial codes were based on prior studies of adolescent and parent attitudes about barriers towards engaging in mental health care and research (Gulliver et al., 2010; Moilanen, 2016). An inductive approach was also used to identify other themes that emerged in the responses. First, the principal investigator and three team members (senior staff member with public health training, a graduate student in public health, and an undergraduate psychology student) read 60 participants' qualitative responses and developed the initial codebook. Then, a training set of 20 participants' responses were coded individually by the three team members. Next, the team refined the codebook based on each coder's analysis of the training set. Each team member then individually coded the rest of the responses in batches of 20 to 40, convening after each batch to check inter-rater reliability, discuss discrepancies, and further refine the codebook before moving on to the next batch. Given the brevity of responses, the unit of text for assignment of codes was the participant's full response to the question. Multiple codes could be applied to each text unit. Final codes and example quotes are shown in Table 3 of the results section. Inter-rater reliability was acceptable, with Krippendorff's alpha ≥0.70 for all codes except the code for parents not wanting their teen to participate in research (Krippendorff's alpha = 0.63).

## 3 Results

### 3.1 Participant characteristics

Demographic and psychiatric characteristics of the 366 adolescent participants recruited via Instagram ads who networked about eating, weight, and/or body image are presented in Table 1. The median age was 16 years (range 15–17). Almost all (95%) participants were female. The majority was non-Hispanic White (65%), while 15% were Hispanic. Participants resided in 44 different states and most (65%) lived in the suburbs. Approximately 85% met clinical or subclinical criteria for an ED, the most common being unspecified feeding or ED (33%) or subclinical bulimia nervosa (31%). Median EDQOL was approximately 1.4 (on a scale from 0 to 4, with higher scores indicating worse quality of life), which is similar to scores from participants in the study that validated the EDQOL (Engel et al., 2006). Among those who met the criteria for a clinical or subclinical ED, only 12% had received any treatment within the last six months. In addition, 64% and 58% screened positive for major depression and GAD, respectively.

**Table 1:** Characteristics of adolescent participants.

Characteristic	*n*^a^ (%)
Age (*n* = 364)	
15 years	107 (29)
16 years	119 (33)
17 years	138 (38)
Gender (*n* = 365)	
Female	346 (95)
Male	2 (1)
Other	17 (5)
Race/ethnicity	
Non-Hispanic White	237 (65)
Non-Hispanic Black	24 (7)
Non-Hispanic other race	49 (13)
Hispanic	56 (15)
Community	
Urban	63 (17)
Suburban	238 (65)
Rural	49 (13)
Not sure	16 (4)
ED diagnosis	
Possible anorexia nervosa	17 (5)
Bulimia nervosa	26 (7)
Binge eating disorder	9 (3)
Subclinical bulimia nervosa	115 (31)
Subclinical binge eating disorder	17 (5)
Purging disorder	4 (1)
Unspecified feeding or eating disorder	122 (33)
At risk	23 (6)
Not at risk	33 (9)
EDQOL (*n* = 331) median (inter-quartile range)	1.36 (0.80, 1.76)
Received treatment in past six months (among *n* = 245 of those screening positive for a ED clinical/subclinical diagnosis)	30 (12)
Major depression^b^ (*n* = 301)	194 (64)
Anxiety disorder^c^ (*n* = 294)	172 (58)

EDQOL = Eating Disorders Quality of Life questionnaire score. Ranges from 0 = never to 4 = always. Higher score indicates worse QOL.

a Total *N* = 366 unless otherwise noted due to missing survey data.

b Score ≥ 10 on PHQ-9.

c Score ≥ 10 on GAD-7.

### 3.2 Willingness to allow researchers to contact parents to obtain their consent for research

**Fig. 1:**
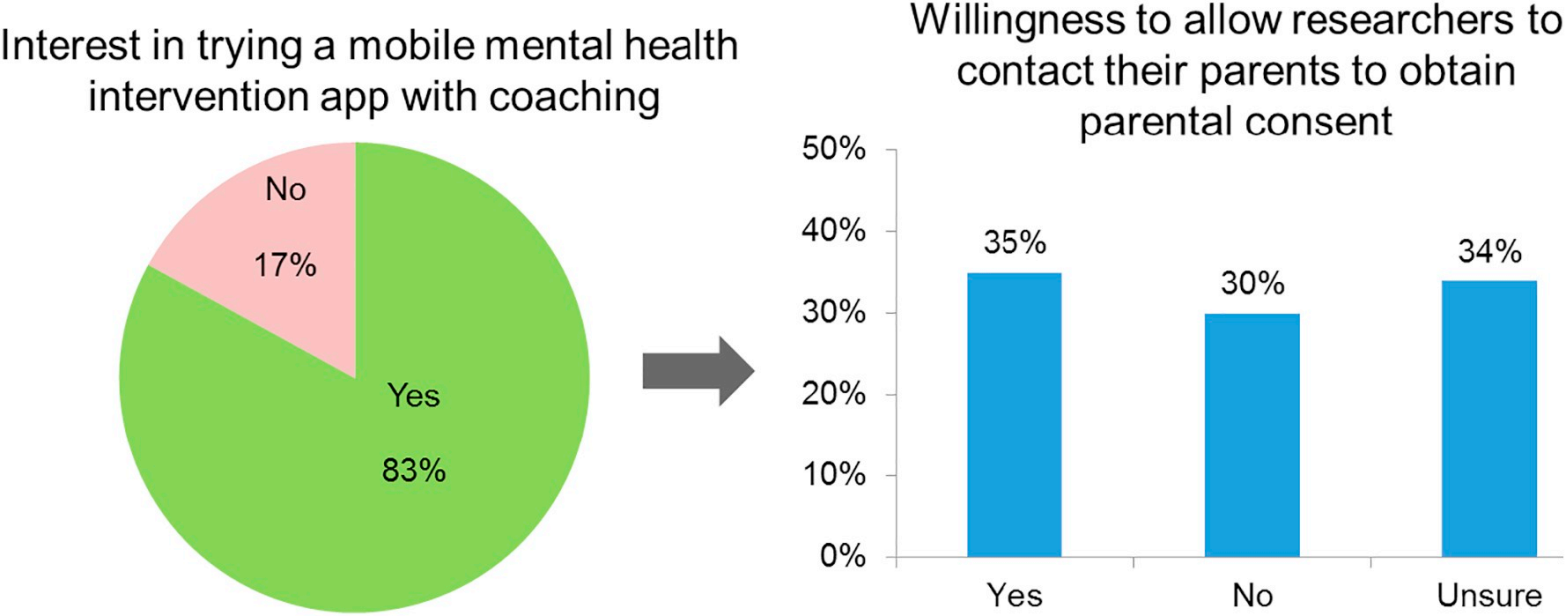
Participants interest in using a mental health app and their willingness to obtain parental consent for research.

Approximately 83% of participants indicated they would be interested in trying a mobile mental health intervention app for EDs that includes e-coaching (Fig. 1). Among those interested, approximately one-third (35%) indicated they would be willing to first allow researchers to contact their parents to obtain parental consent. Of those willing, 51% of participants preferred researchers contacting their parents via email only, 13% preferred phone only, and 37% were comfortable with phone or email contact. Approximately 30% were not willing to allow researchers to contact their parents, and another 34% were unsure.

Differences between participants willing to allow researchers to contact their parents to obtain parental consent, those who are unwilling, and those who are unsure about allowing researchers to do so are shown in Table 2. There are significant differences between white participants and participants of other ethnicities/races, *(p* = 0.009), participants with and without depression *(p* = 0.005), participants with and without anxiety *(p* = 0.024), and people with higher total EDQOL scores *(p* = 0.003). Specifically, white participants were more likely to be unwilling than unsure to allow researchers to contact their parents to obtain parental consent compared with participants of other ethnicities/races (77% vs. 64%, *p* = 0.002). Participants suffering from depression were more likely to be willing and unwilling than unsure to allow researchers to contact their parents to obtain parental consent compared with those without depression (64% vs.59% vs. 44%, *p* = 0.003 for willing vs. unsure, *p* = 0.016 for unwilling vs. unsure). Participants suffering from anxiety were more likely to be willing than unsure to allow researchers to contact their parents to obtain parental consent compared with those without anxiety (56% vs.39%, *p* = 0.007).

**Table 2:** Differences between adolescents willing, unwilling, and unsure about allowing researchers to contact their parents to obtain consent.

Participant characteristic	Willing	Unwilling	Unsure	*p*	Pairwise comparisons
	n (%)				
15 years old 16 years old 17 years old	28 (27) 32 (30) 45 (43)	28 (30) 29 (32) 35 (38)	32 (31) 37 (36) 35 (34)	0.738	all *p* > 0.05
Race - White	60 (57)	71 (77)	67 (64)	0.009	Unwilling > Willing (*p* = 0.002)
Major depression	64 (75)	59 (71)	44 (53)	0.005	Willing (*p* = 0.003), Unwilling (*p* = 0.016) > Unsure
Anxiety disorder	56 (69)	49 (60)	39 (48)	0.024	Willing > Unsure (*p* = 0.007)

	median (IQR)				

EDQOL	1.56 (1.00-1.88)	1.46 (0.80-1.80)	1.08 (0.60-1.48)	0.003	Willing > Unsure (*p* = 0.002)

EDQOL = Eating Disorders Quality of Life questionnaire score. Ranges from 0 = never to 4 = always. Higher score indicates worse QOL.

### 3.3 Reasons for unwillingness or uncertainty about obtaining parental consent

Among the 196 participants who were uncertain about or unwilling to allow researchers to contact their parents to obtain parental consent, 166 answered open-ended questions asking further explanation. Table 3 presents reasons, or themes, commonly noted by these respondents along with some example responses. The most common reason for participants' unwillingness to allow researchers to contact their parents for consent purposes was the importance of retaining their privacy, noted by one-third (33%) of participants. In addition, nearly one-quarter (23%) felt that their parents lacked awareness or understanding about mental health wellness, in general, or the issues that the teen was facing. Twenty percent of participants were worried about the reaction that their parents might have regarding researchers contacting them to obtain consent for their child to participate in an ED study, and 16% felt that they were autonomous and did not need their parents' input or permission. Some less common reasons included a poor relationship with their parents (10%), that parents would not want their teen to participate in research (8%), and concerns about online security (2%).

**Table 3:** Reasons teens were unwilling or uncertain about allowing researchers to contact their parents for consent purposes (*N* = 166).

Theme	N (%)	Example responses
Teen privacy	55 (33)	● “They don't know about my problem and I want to keep it that way.” ● “I'm not open about my issues with my parents”
Parents lack an understanding of mental health or are unaware of the problem	38 (23)	● “I am not sure they would approve, seeing as they don't necessarily believe in mental health problems. Also, mental health is stigmatized in my family.” ● “They don't know about my eating disorder”
Teen is concerned about parents' reaction/response	34 (20)	● “I don't want them to worry more about me” ● “They would be concerned/annoyed that I've never talked to them about this before”
Teen autonomy	26 (16)	● “I do not like or trust my parents and I don't want them involved in this” ● “I feel old enough to make my own decisions about apps I'd like to try out”
Poor parent-teen relationship	17 (10)	● “My family would never let me get help. A lot of parents don't care about whether their kid is hurting or not. This won't work if you make people tell their families. It just won't”. ● “My parents can be judgmental at some points so I don't want them to tell me something about wanting to try something new”.
Parent would not want the teen to participate in research	13 (8)	● “They might think it's a scam and say no” ● “My parents might not be open to me participating in such a study or understanding of how I might benefit from such an app”
Fears about online security/privacy	3 (2)	● “I am not sure that this survey is really trustworthy or that they would want me taking it” ● “I do not know what kind of information they will be collecting, so I am undecided”

## 4 Discussion

Despite the severity of EDs and the need for early intervention, a wide treatment gap exists for adolescents with ED symptoms. Therefore, it is highly relevant that majority of our underage participants (83%), most of whom tended to meet clinical or subclinical criteria for an ED, had interest in trying a mobile mental health intervention for the treatment of EDs. Digital therapeutic tools (e.g., mobile applications) have been shown to be accessible and low-cost interventions and our results of participant interest in an mHealth intervention for ED treatment strongly support the potential for their use among adolescents who struggle with EDs, are not currently engaged in treatment, and could likely benefit from an intervention. However, among adolescents who indicated interest, only one-third (35%) expressed willingness to allow researchers to contact their parents to obtain parental consent, while nearly two-thirds felt unwilling (30%) or unsure (34%). Thus, although adolescents with EDs express high interest in using a mobile app-based intervention, the obtainment of parental consent could be a potential barrier for future research studies of these novel interventions.

Results further indicated that certain subgroups of participants, including those with more severe manifestations of illness, were more willing to allow researchers to contact their parents to obtain parental consent. Specifically, these participants endorsed worse ED-related quality of life and suffered from comorbid major depression and anxiety. Previous research found that mental disorder severity is strongly related to service utilization among adolescents, suggesting that more severe symptomatology potentially drives mental health treatment seeking among adolescents (Merikangas et al., 2011). In the context of our findings, there was an association between a greater adolescent willingness to allow researchers to contact their parents to obtain parental consent and more severe ED psychopathology and lower quality of life. These findings suggest that teens with EDs may be more compelled to involve their parents when their mental illness symptoms are more pronounced. Supporting the relationship between ED severity and willingness to allow researchers to contact their parents to obtain parental consent, we also found that individuals at risk for an ED were less willing than those with a clinical/subclinical ED to allow researchers to contact their parents to obtain parental consent. This latter finding is particularly concerning given the importance of early intervention for EDs.

Adolescence is a crucial time for early assessment and intervention surrounding mental health, given that three quarters of all lifetime mental disorders have their primary onset during this developmental period (Kessler et al., 2007; Kessler et al., 2005), yet these disorders often go untreated due to lack of help-seeking and health literacy among this population (Rickwood et al., 2005). Taking into account the prevalent use of cell phones among adolescents, mHealth presents a promising method of both intervention and prevention for adolescents (Seko et al., 2014). Previous studies have shown that an online self-help program for anxiety prevention, intervention, or early treatment purposes that did not require parental consent for adolescents has demonstrated moderate to high program acceptability when delivered this way (March et al., 2018). Thus, future research studies of mental health digital therapeutics requiring parental consent may fail to capture ‘at risk’ adolescents because of their greater unwillingness to allow researchers to contact their parents to obtain parental consent, allowing the illness to progress in severity and having negative consequences on their health and wellbeing.

Additionally, participants who identified as non-White ethnic and racial minorities were also more willing to allow researchers to contact their parents to obtain parental consent (44%) than their White counterparts (30%). This may be due to interest in digital therapeutics for this subgroup and/or increased comfort with discussing mental health topics with parents versus White counterparts. This is consistent with previous studies that have found non-White adolescents express a greater belief that their parents/guardians would support cell phone–based communication with a health care provider compared to White adolescents (Sawni et al., 2017). Notably, among adolescents with mood disorders, Hispanics and non-Hispanic Blacks are much less likely to receive mental health treatment and there is clear need for evidence-based behavioral or biomedical prevention or treatment programs targeted to non-White ethnic and racial minorities, but few such programs exist, due in large part to scant research knowledge on how to develop and tailor efforts towards their needs (Merikangas et al., 2011). Even more pertinent to our study, individuals from racial and ethnic minority backgrounds with EDs are significantly less likely than their White counterparts to be diagnosed with an ED, receive care or a referral for further evaluation, or even be asked by a doctor about ED symptoms (Marques et al., 2011). However, minority populations, including those who identify as African American and Hispanic, are using mobile phones to access health information more frequently than those who classify themselves as White (Anderson-Lewis et al., 2018). Additionally, the potential of digital therapeutics is especially significant for younger populations, as they have a high level of cell phone use across diverse sociodemographic domains (Koivusilta et al., 2007). This provides a unique opportunity to reach young, underserved populations and exceed barriers to health care access via mHealth interventions. Further, given the current limited understanding of how to facilitate safe and fair access to ED-focused health research and interventions for overlooked vulnerable subpopulations, the willingness of adolescents of racial and ethnic minorities to allow researchers to contact their parents to obtain parental consent for research involvement offers the promise of identifying an effective digital intervention to address this gap.

Adolescents commonly cited their desire for privacy, feeling that parents lacked an understanding of mental health or were unaware of the problem, and concerns about their parents' reaction if they were approached for the obtainment of parental consent. Parallel themes have been identified as reasons why adolescents do not seek help for mental illnesses. Indeed, a systematic review identified a preference for self-reliance, issues related to confidentiality and trust, and stigma as key barriers to mental health help-seeking in young people (Gulliver et al., 2010). Therefore, obtaining a parental consent waiver may aid in protecting adolescent privacy and confidentiality and as a result, increase adolescent engagement in ED and mental health treatment in general. However, if it is determined that parental consent is a necessary requirement to protect subjects and a waiver is not permitted for certain studies, taking steps to motivate adolescents and ensure them that their privacy and autonomy will be protected may increase their engagement. Further, the utilization of a cell phone can provide adolescents with “sufficient privacy to support sensitive therapeutic activities,” and this capacity of mobile phones to offer user privacy is considered to increase levels of perceived autonomy, control, and self-esteem in young people (Kauer et al., 2012). Given the personal nature of cell phones, a mobile app intervention may be successful in promoting self-monitoring strategies that aid adolescents in becoming aware of their mental health and developing coping strategies.

Self-paced mobile application interventions can provide psychoeducation to adolescents about the importance of early intervention for mental health symptoms, addressing stigma-related concerns, and equipping adolescents with strategies for approaching their parents about concerns and the need for intervention. Additionally, increasing parents' ability to recognize adolescent distress and to respond in ways that successfully result in help, whether that it be through digital or in-person resources, may facilitate the use of services among adolescents with EDs or other mental illnesses (Logan and King, 2006).

Results from this study suggest that the majority of adolescents socially networking about eating and/or body image expressed interest in using mobile mental health interventions to address their ED symptoms. Given that such novel tools have the potential to address ED and general mental health treatment gaps among adolescents, there is a need to better understand how to address parental consent as a potential barrier. It may be that an improved description on the extent of and reason for parental involvement (e.g., explicitly stating that plans for self-harm would initiate parent contact but that other information the adolescent shared would be kept private) and/or evaluating different language options (for e.g., testing ways to motivate adolescents to involve parents for providing consent) would result in more adolescents indicating their willingness to allow researchers to contact their parents to obtain consent. Given the limited science in this topic area, we believe that continued refinement in the approach to improve teens' willingness to allow researchers to contact their parents to obtain their consent is a timely and significant research area.

The findings of this study should be interpreted in the context of limitations. Findings are based on an online self-reported questionnaire and could be subject to social desirability bias. Additionally, our results may not be entirely representative of the adolescent ED population as this study excluded individuals younger than 15 years and younger adolescents are also affected by EDs. Nevertheless, given the importance of early interventions for older adolescents with disordered eating, teens aged 15 to 17 serve as an appropriate target for the early identification and treatment of at-risk individuals.

Despite limitations, there is scant intervention research on adolescents with EDs. Parental consent can be a barrier to recruitment and participation of underage teens in research and lead to narrow treatment options for this vulnerable and important subgroup. Informed by this current study, future studies should strive to pilot approaches for overcoming the potential parental consent barrier to facilitate teen involvement in accessible digital interventions and research in general. Targeting this barrier will not only help create efficient methods to successfully engage adolescents in research, but will also allow future studies to tailor and adapt interventions among adolescents to facilitating their engagement in research and treatments that are designed specifically for them. Further, identifying teens that report they are “unsure” about engaging in research due to parental consent requirements may offer a promising group to further assess motivations towards engagement in both digital and in-person treatment. Especially in light of the persistent treatment gap for adolescents with EDs and the critical importance of early intervention, piloting strategies for engaging underage adolescents and their parents in research of a scalable, cost-effective mobile intervention is of the utmost importance.

## Funding

This study was funded by the 10.13039/100000002National Institutes of Health [grant number K02 DA043657] and the 10.13039/100000025National Institute of Mental Health [grant numbers R21 MH112331, R34 MH119170-01].

## Declaration of competing interest

The authors have no conflicts of interest to declare.

## References

[bb0005] Anderson-Lewis C., Darville G., Mercado R.E., Howell S., Di Maggio S. (2018). mHealth technology use and implications in historically underserved and minority populations in the United States: systematic literature review. JMIR mHealth uHealth.

[bb0010] Balen R., Blyth E., Calabretto H., Fraser C., Horrocks C., Manby M. (2006). Involving children in health and social research: ‘human becomings’ or ‘active beings’?. Childhood.

[bb0015] Bauermeister J., Pingel E., Zimmerman M., Couper M., Carballo-Dieguez A., Strecher V.J. (2012). Data quality in web-based HIV/AIDS research: handling invalid and suspicious data. Field Methods.

[bb0020] Berry R.R., Lai B. (2014). The emerging role of technology in cognitive–behavioral therapy for anxious youth: a review. J. Ration. Emot. Cogn. Behav. Ther..

[bb0025] Braun V., Clarke V. (2006). Using thematic analysis in psychology. Qual. Res. Psychol..

[bb0030] Crane S., Broome M.E. (2017). Understanding ethical issues of research participation from the perspective of participating children and adolescents: a systematic review. Worldviews Evid.-Based Nurs..

[bb0035] Das J.K., Salam R.A., Lassi Z.S., Khan M.N., Mahmood W., Patel V., Bhutta Z.A. (2016). Interventions for adolescent mental health: an overview of systematic reviews. J. Adolesc. Health.

[bb0040] D’Souza C.M., Forman S.F., Austin S.B. (2005). Follow-up evaluation of a high school eating disorders screening program: knowledge, awareness and self-referral. J. Adolesc. Health.

[bb0045] Ebert D.D., Zarski A.-C., Christensen H., Stikkelbroek Y., Cuijpers P., Berking M., Riper H. (2015). Internet and computer-based cognitive behavioral therapy for anxiety and depression in youth: a meta-analysis of randomized controlled outcome trials. PLoS One.

[bb0050] Engel S.G., Wittrock D.A., Crosby R.D., Wonderlich S.A., Mitchell J.E., Kolotkin R.L. (2006). Development and psychometric validation of an eating disorder-specific health-related quality of life instrument. Int J Eat Disord.

[bb0055] Fenner Y., Garland S.M., Moore E.E., Jayasinghe Y., Fletcher A., Tabrizi S.N., Wark J.D. (2012). Web-based recruiting for health research using a social networking site: an exploratory study. J. Med. Internet Res..

[bb0060] Fitzsimmons-Craft E.E., Firebaugh M.L., Graham A.K., Eichen D.M., Monterubio G.E., Balantekin K.N., Wilfley D.E. (2019). State-wide university implementation of an online platform for eating disorders screening and intervention. Psychol. Serv..

[bb0065] Forrest L.N., Smith A.R., Swanson S.A. (2017). Characteristics of seeking treatment among US adolescents with eating disorders. Int. J. Eat. Disord..

[bb0070] Graham A.K., Trockel M., Weisman H., Fitzsimmons-Craft E.E., Balantekin K.N., Wilfley D.E., Taylor C.B. (2018). A screening tool for detecting eating disorder risk and diagnostic symptoms among college-age women. J. Am. Coll. Heal..

[bb0075] Gulliver A., Griffiths K.M., Christensen H. (2010). Perceived barriers and facilitators to mental health help-seeking in young people: a systematic review. BMC Psychiatry.

[bb0080] Hollis C., Falconer C.J., Martin J.L., Whittington C., Stockton S., Glazebrook C., Davies E.B. (2017). Annual research review: digital health interventions for children and young people with mental health problems–a systematic and meta-review. J. Child Psychol. Psychiatry.

[bb0085] Kauer S.D., Reid S.C., Crooke A.H.D., Khor A., Hearps S.J.C., Jorm A.F., Patton G. (2012). Self-monitoring using mobile phones in the early stages of adolescent depression: randomized controlled trial. J. Med. Internet Res..

[bb0090] Kazdin A.E. (2019). Annual research review: expanding mental health services through novel models of intervention delivery. J. Child Psychol. Psychiatry.

[bb0095] Kazdin A.E., Fitzsimmons-Craft E.E., Wilfley D.E. (2017). Addressing critical gaps in the treatment of eating disorders. Int J Eat Disord.

[bb0100] Kessler R.C., Berglund P., Demler O., Jin R., Merikangas K.R., Walters E.E. (2005). Lifetime prevalence and age-of-onset distributions of DSM-IV disorders in the National Comorbidity Survey Replication. Arch. Gen. Psychiatry.

[bb0105] Kessler R.C., Amminger G.P., Aguilar-Gaxiola S., Alonso J., Lee S., Ustün T.B. (2007). Age of onset of mental disorders: a review of recent literature. Current Opinion in Psychiatry.

[bb0110] Klump K.L., Bulik C.M., Kaye W.H., Treasure J., Tyson E. (2009). Academy for eating disorders position paper: eating disorders are serious mental illnesses. Int J Eat Disord.

[bb0115] Koivusilta L.K., Lintonen T.P., Rimpela A.H. (2007). Orientations in adolescent use of information and communication technology: a digital divide by sociodemographic background, educational career, and health. Scand J Public Health.

[bb0120] Kroenke K., Spitzer R.L., Williams J.B. (2001). The PHQ-9: validity of a brief depression severity measure. J. Gen. Intern. Med..

[bb0125] Kroenke K., Spitzer R.L., Williams J.W., Monahan P.O., Löwe B. (2007). Anxiety disorders in primary care: prevalence, impairment, comorbidity, and detection. Ann. Intern. Med..

[bb0130] Leiner D.J. (2013). Too fast, too straight, too weird: Post hoc identification of meaningless data in internet surveys. Surv. Res. Methods.

[bb0135] Liu C., Cox R.B., Washburn I.J., Croff J.M., Crethar H.C. (2017). The effects of requiring parental consent for research on adolescents’ risk behaviors: a meta-analysis. J. Adolesc. Health.

[bb0140] Logan D.E., King C.A. (2006). Parental facilitation of adolescent mental health service utilization: a conceptual and empirical review. Clin. Psychol. Sci. Pract..

[bb0145] Malbon K., Romo D. (2013). Is it ok 2 txt? Reaching out to adolescents about sexual and reproductive health. Postgrad. Med. J..

[bb0150] March S., Spence S.H., Donovan C.L., Kenardy J.A. (2018). Large-scale dissemination of internet-based cognitive behavioral therapy for youth anxiety: feasibility and acceptability study. J. Med. Internet Res..

[bb0155] Marques L., Alegria M., Becker A.E., Chen C.n., Fang A., Chosak A., Diniz J.B. (2011). Comparative prevalence, correlates of impairment, and service utilization for eating disorders across US ethnic groups: implications for reducing ethnic disparities in health care access for eating disorders. Int. J. Eat. Disord..

[bb0160] Merikangas K.R., He J.P., Burstein M., Swendsen J., Avenevoli S., Case B., Olfson M. (2011). Service utilization for lifetime mental disorders in US adolescents: results of the National Comorbidity Survey–Adolescent Supplement (NCS-A). J. Am. Acad. Child Adolesc. Psychiatry.

[bb0165] Militello L.K., Kelly S.A., Melnyk B.M. (2012). Systematic review of text-messaging interventions to promote healthy behaviors in pediatric and adolescent populations: implications for clinical practice and research. Worldviews Evid.-Based Nurs..

[bb0170] Modan-Moses D., Yaroslavsky A., Novikov I., Segev S., Toledano A., Miterany E., Stein D. (2003). Stunting of growth as a major feature of anorexia nervosa in male adolescents. Pediatrics.

[bb0175] Moilanen K.L. (2016). Why do parents grant or deny consent for adolescent participation in sexuality research?. Journal of Youth and Adolescence.

[bb0180] Murray E., Hekler E.B., Andersson G., Collins L.M., Doherty A., Hollis C., Wyatt J.C. (2016). Evaluating digital health interventions: key questions and approaches.

[bb0185] Olmos J.M., Valero C., del Barrio A.G., Amado J.A., Hernandez J.L., Menendez-Arango J., Gonzalez-Macias J. (2010). Time course of bone loss in patients with anorexia nervosa. Int J Eat Disord.

[bb0190] Reyes-Rodríguez M.L., Franko D.L., Matos-Lamourt A., Bulik C.M., Von Holle A., Cámara-Fuentes L.R., Suárez-Torres A. (2010). Eating disorder symptomatology: prevalence among Latino college freshmen students. J. Clin. Psychol..

[bb0195] Rickwood D., Deane F.P., Wilson C.J., Ciarrochi J. (2005). Young people’s help-seeking for mental health problems. Australian e-Journal for the Advancement of Mental Health.

[bb0200] Rojas N.L., Sherrit L., Harris S., Knight J.R. (2008). The role of parental consent in adolescent substance use research. J. Adolesc. Health.

[bb0205] Rooksby M., Elouafkaoui P., Humphris G., Clarkson J., Freeman R. (2015). Internet-assisted delivery of cognitive behavioural therapy (CBT) for childhood anxiety: systematic review and meta-analysis. Journal of Anxiety Disorders.

[bb0210] Saekow J., Jones M., Gibbs E., Jacobi C., Fitzsimmons-Craft E.E., Wilfley D., Taylor C.B. (2015). StudentBodies-eating disorders: a randomized controlled trial of a coached online intervention for subclinical eating disorders. Internet Interv..

[bb0215] Sawni A., Cederna-Meko C., LaChance J.L., Buttigieg A., Le Q., Nunuk I., Burrell K.M. (2017). Feasibility and Perceptions of Cell Phone–Based, Health-Related Communication With Adolescents in an Economically Depressed Area. Clin. Pediatr..

[bb0220] Schnall R., Bakken S., Rojas M., Travers J., Carballo-Dieguez A. (2015). mHealth technology as a persuasive tool for treatment, care and Management of Persons Living with HIV. AIDS Behav..

[bb0225] Seko Y., Kidd S., Wiljer D., McKenzie K. (2014). Youth mental health interventions via Mobile phones: a scoping review. Cyberpsychol. Behav. Soc. Netw..

[bb0230] Spitzer R.L., Kroenke K., Williams J.W., Löwe B. (2006). A brief measure for assessing generalized anxiety disorder: the gad-7. Arch. Intern. Med..

[bb0235] Stice E., Marti C.N., Rohde P. (2013). Prevalence, incidence, impairment, and course of the proposed DSM-5 eating disorder diagnoses in an 8-year prospective community study of young women. J. Abnorm. Psychol..

[bb0240] Swanson S.A., Crow S.J., Le Grange D., Swendsen J., Merikangas K.R. (2011). Prevalence and correlates of eating disorders in adolescents: results from the national comorbidity survey replication adolescent supplement. Arch. Gen. Psychiatry.

[bb0245] Vigerland S., Lenhard F., Bonnert M., Lalouni M., Hedman E., Ahlen J., Ljótsson B. (2016). Internet-delivered cognitive behavior therapy for children and adolescents: a systematic review and meta-analysis. Clin. Psychol. Rev..

[bb0250] Volpe U., Tortorella A., Manchia M., Monteleone A.M., Albert U., Monteleone P. (2016). Eating disorders: what age at onset?. Psychiatry Res..

[bb0255] Wartella E., Rideout V., Montague H., Beaudoin-Ryan L., Lauricella A. (2016). Teens, health and technology: a national survey. Media Commun..

[bb0260] Ybarra M.L., Prescott T.L., Philips G.L., Bull S.S., Parsons J.T., Mustanski B. (2016). Iteratively developing an mHealth HIV prevention program for sexual minority adolescent men. AIDS Behav..

